# Antiplatelet Activity of *Morus alba* Leaves Extract, Mediated via Inhibiting Granule Secretion and Blocking the Phosphorylation of Extracellular-Signal-Regulated Kinase and Akt

**DOI:** 10.1155/2014/639548

**Published:** 2014-02-19

**Authors:** Dong-Seon Kim, Hyun Dong Ji, Man Hee Rhee, Yoon-Young Sung, Won-Kyung Yang, Seung Hyung Kim, Ho-Kyoung Kim

**Affiliations:** ^1^Basic Herbal Medicine Research Group, Korea Institute of Oriental Medicine, Daejeon 305-811, Republic of Korea; ^2^College of Veterinary Medicine, Kyungpook National University, Daegu 702-701, Republic of Korea; ^3^Institute of Traditional Medicine & Bioscience, Daejeon University, Daejeon 300-716, Republic of Korea

## Abstract

*Ethnopharmacological Relevance*. *Morus alba* L. leaves (MAE) have been used in fork medicine for the treatment of beriberi, edema, diabetes, hypertension, and atherosclerosis. However, underlying mechanism of MAE on cardiovascular protection remains to be elucidated. Therefore, we investigated whether MAE affect platelet aggregation and thrombosis. *Materials and Methods*. The anti-platelet activity of MAE was studied using rat platelets. The extent of anti-platelet activity of MAE was assayed in collagen-induced platelet aggregation. ATP and serotonin release was carried out. The activation of integrin **α**_IIb_ **β**_3_ and phosphorylation of signaling molecules, including MAPK and Akt, were investigated with cytofluorometer and immunoblotting, respectively. The thrombus formation *in vivo* was also evaluated in arteriovenous shunt model of rats. *Results*. HPLC chromatographic analysis revealed that MAE contained rutin and isoquercetin. MAE dose-dependently inhibited collagen-induced platelet aggregation. MAE also attenuated serotonin secretion and thromboxane A_2_ formation. In addition, the extract *in vivo* activity showed that MAE at 100, 200, and 400 mg/kg significantly and dose-dependently attenuated thrombus formation in rat arterio-venous shunt model by 52.3% (*P* < 0.001), 28.3% (*P* < 0.01), and 19.1% (*P* < 0.05), respectively. *Conclusions*. MAE inhibit platelet activation, TXB2 formation, serotonin secretion, aggregation, and thrombus formation. The plant extract could be considered as a candidate to anti-platelet and antithrombotic agent.

## 1. Introduction

Cardiovascular diseases, including thrombosis, stroke, ischemic, and coronary heart diseases, are a leading cause of mortality, accounting for around 30% of global deaths [1]. Especially, thrombotic diseases constitute a major cardiovascular complication affecting a great number of patients. Thrombosis is closely related to activated platelet adhesion, aggregation, secretion functions, and activation of intrinsic and extrinsic coagulation systems, which cause blood coagulation and fibrin formation [2]. Most acute coronary syndromes are caused by platelet aggregation and subsequent thrombus formation in areas of ruptured atheromatous plaques [3, 4]. Therefore, inhibiting platelet function represents a promising approach for preventing thrombosis [5, 6].

Antiplatelet drugs have been developed to inhibit platelet activity in acute thrombotic situations as well as to prevent adverse events and treatment of atherothrombotic disease [7]. Aspirin and clopidogrel for oral administration and glycoprotein IIa/IIIb antagonists (abciximab, eptifibatide, tirofiban, etc.) for injection are commonly used antiplatelet drugs, but they have several clinical disadvantages including gastrointestinal side-effects, hemorrhage and thrombocytopenia [7–11]. With this regards, much attention has been given to the development of dietary supplements or herbal medicines for prevention or treatment of cardiovascular diseases for their merits in safety [12].

*Morus alba* L. (Mulberry) leaf belongs to the Moraceae family, distributed mainly in the temperate and subtropical regions in the northern hemisphere. It has been traditionally used in China, Korea, Japan, and other Asian countries as herbal tea as well as herbal medicine. According to the oldest Korean medical book (Dong-ui-bo-gam), it is called “Sang-Yeop” and introduced to be beneficial in alleviating beriberi, edema and pains [13].

Recent studies have reported that it shows antiatherosclerosis [14], antihypertension [15, 16], antiobesity [17], antidiabetic [18, 19], liver protection [20], antiviral [21] and antimicrobial [22] effects. This antiplatelet or antithrombotic effect of *M. alba* leaves has never been described before, though an antiplatelet effect has been described with its barks [23].

In this study, we discovered that the *M. alba* leaves extract has strong inhibitory activities on collagen-induced platelet aggregation *in vitro* and on thrombosis *in vivo*.

## 2. Materials and Methods

### 2.1. Materials

Collagen was obtained from Chrono-Log Co. (Havertown, PA, USA). Aspirin, fibrinogen, plasmin and dimethyl sulfoxide (DMSO) were from Sigma Chemical Co. (St. Louis, MO, USA). Fura-2/AM was obtained from Sigma Chemical Co. (St. Louis, MO, USA). Antibodies against phospho-p44/42, p44/42, phospho-p38, p38, phospho-SAPK/JNK, phospho-PI3K (p85), phospho-Akt, and *β*-actin were acquired from Cell Signaling (Beverly, MA, USA). ATP assay kits were purchased from Biomedical Research Service Center (Buffalo, NY, USA). A TXB_2_ enzyme immunoassay (EIA) kit was from Cayman Chemical (Ann Arbor, MI, USA). Fibrinogen Alexa Fluor 488 conjugate was obtained from Molecular Probes (Eugene, OR, USA). HPLC-grade reagents, methanol and water were obtained from J. T. Baker (Phillipsburg, NJ, USA). All other chemicals were of reagent grade.

### 2.2. Preparation of the Ethanol Extract and Solvent Fractions from *Morus alba*

*Morus alba *leaves as a dried herb was purchased from Omniherb Co. (Yeongcheon, Korea) and was authenticated, based on its microscopic and macroscopic characteristics, by the Classification and Identification Committee of the Korea Institute of Oriental Medicine (KIOM). The committee consisted of nine experts in the fields of plant taxonomy, botany, pharmacognosy, and herbology. A voucher specimen (MAL-20120605) was deposited at the herbarium of the Department of Herbal Resources Research at the KIOM.

Dried leaves of *Morus alba* (500 g) were extracted twice with 70% (v/v) ethanol (5 L) for 4 h under mantle-reflux. The extracts were filtered and evaporated under reduced pressure to give *Morus alba* leaves extract (MAE, 56.0 g). The extract (40 g) was suspended in water (1.2 L) to be partitioned subsequently with *n*-hexane (2 × 1.2 L), ethyl acetate (2 × 1.2 L), and then *n*-butanol (2 × 1.2 L) and the solvent-soluble fractions were evaporated to afford hexane- (3.9 g), ethyl acetate- (1.8 g), butanol- (6.7 g) and water-soluble (27.6 g) fraction, respectively.

The extract and the fractions were dissolved in saline or phosphate-buffered saline (PBS) and then filtered through a 0.22 m syringe filter for experiments.

### 2.3. High Performance Liquid Chromatography Analysis

The sample was analyzed by reverse phase-high performance liquid chromatography using Waters Alliance 2695 system (Waters Co., Milford, MA, USA), coupled with a 2996 photodiode array detector. Data processing was carried out using Empower software (Waters Co.). Phenomenex Luna C18 column (250 mm × 4.6 mm; particle size 5 *μ*m, Phenomenex, Torrance, CA, USA) was used as stationary phase. The mobile phase consisted of eluent A (0.1% trifluoroacetic in water) and eluent B (acetonitrile). The starting eluent was 90% A and 10% B. The proportion of eluent B was increased linearly to 40% from 10 min to 40 min. The column temperature was kept at 40°C and the detector wavelength was set over the range of 190 to 400 nm and recorded at 360 nm. The flow rate was 1.0 mL/min, and the injection volume was 10 *μ*L, and the column temperature was kept at 40°C.

Identification was based on retention time, UV spectra by comparison with commercial standards. For each compound, peak areas were determined at the wavelength providing maximal UV absorbance. Calibration curves of the standards ranging from 12.5 to 200 *μ*g/mL (5 levels) revealed good linearity with *R* ^2^ values exceeding 0.99 (peak areas versus concentration). Quantitation was performed based on external standards with a mixture of standards of known concentration that were analyzed in duplicate before and after the batch of samples, and the peak areas were used to calculate the sample contents of the compounds.

### 2.4. Experimental Animals

Male Sprague Dawley rats (300~350 g) were purchased from Japan SLC (Hamamatsu, Japan). The animals were acclimated for 1 week prior to the experiments, and housed in an air-conditioned animal room with a 12/12 h light/dark cycle at a temperature of 22 ± 1°C and humidity of 50 ± 10%. The animals were provided with a laboratory diet and water *ad libitum*.

All experimental protocols involving the use of animals were conducted in accordance with National Institutes of Health (NIH) guidelines and approved by the Committee on Animal Care at the Korea Institute of Oriental Medicine.

### 2.5. Washed Rat Platelet Preparation

Blood was withdrawn from abdominal vein of rats and collected directly into anticoagulant citrate dextrose (ACD) solution that contained 0.8% citric acid, 2.2% trisodium citrate, and 2% dextrose (w/v). Washed platelets were prepared as previously described [24]. Briefly, platelet-rich plasma (PRP) was obtained by centrifuging rabbit blood samples at 230 ×g for 10 min. Platelets were precipitated by centrifugation of the PRP at 800 ×g for 15 min and washed with HEPES buffer (137 mM NaCl, 2.7 mM KCl, 1 mM MgCl_2_, 5.6 mM glucose, 3.8 mM HEPES, and pH 6.5) containing 0.35% BSA and 0.4 mM EGTA. The washed platelets were resuspended in HEPES buffer (pH 7.4) and the cell dilution was adjusted to 4 × 10^8^ cells/mL.

### 2.6. Platelet Aggregation *In Vitro* Assay

Platelet aggregation was evaluated as previously described [25]. Aggregation was monitored by measuring light transmission with an aggregometer (Chrono-Log, Havertown, PA, USA). The washed platelets (3 × 10^8^/mL) were pre-incubated at 37°C for 2 min with either MAE or vehicle and then stimulated with 2.5 *μ*g/mL collagen. The mixture was further incubated for 5 min with stirring at 170 ×g. The vehicle concentration was less than 0.1% to minimize the effect of this reagent.

### 2.7. [Ca^2+^]_*i*_ Measurement

The intracellular calcium ion concentration ([Ca^2+^]_*i*_) was measured with Fura-2/AM as previously described [26]. Briefly, the platelets were incubated with 5 *μ*M of Fura-2/AM for 30 min at 37°C and washed. The Fura-2-loaded platelets (3 × 10^8^/mL) were then pre-incubated with MAE for 2 min at 37°C in the presence of 1 mM CaCl_2_ and subsequently stimulated with collagen for 5 min. Fluorescent signals were recorded using a Hitachi F-2500 fluorescence spectrofluorometer (F-2500, Hitachi, Japan). Light emission was measured at 510 nm with simultaneous excitation at 340 and 380 nm that changed every 0.5 s. Fura-2 fluorescence in the cytosol measured with the spectrofluorometer was calculated as previously described by Schaeffer and Blaustein [27] with the following formula: [Ca^2+^]_*i*_ 224 nM ×  (*F* − *F*_min⁡_)/(*F*_max⁡_ − *F*), in which 224 nM is the dissociation constant of the Fura-2-Ca^2+^ complex, and *F*_min⁡_ and *F*_max⁡_ represent the fluorescence intensity levels at very low and very high Ca^2+^ concentrations, respectively. In our experiment, *F*_max⁡_ was the intensity of the Fura-2-Ca^2+^ complex fluorescence at 510 nm after the platelet suspension containing 1 mM of CaCl_2_ had been solubilized with Triton X-100 (0.1%). *F*_min⁡_ was the fluorescence intensity of the Fura-2-Ca^2+^ complex at 510 nm after the platelet suspension containing 20 mM Tris/3 mM of EGTA had been solubilized with Triton X-100 (0.1%). *F* represented the intensity of Fura-2 complex fluorescence at 510 nm after the platelet suspension was stimulated with collagen with or without MAE in the presence of 1 mM CaCl_2_.

### 2.8. ATP Release Assay

Washed platelets (3 × 10^8^/mL) were pre-incubated for 2 min at 37°C with various concentrations of MAE and then stimulated with 2.5 *μ*g/mL collagen. After the reaction was terminated, the cells were centrifuged and the supernatants were used for the assay. ATP release was measured in a luminometer (GloMax 20/20; Promega, Madison, USA) using an ATP assay kit (Biomedical Research Service Center, Buffalo, NY, USA) according to manufacturer's instructions.

### 2.9. Measurement of Serotonin Secretion

The washed platelets (3 × 10^8^/mL) were pre-incubated for 2 min at 37°C with various concentrations of MAE in the presence of 1 mM of Ca^2+^. The reaction mixture was further incubated with collagen (2.5 *μ*g/mL) for 5 min. After terminating the aggregation reaction, the mixture was immediately centrifuged at 12,000 ×g for 5 min at 4°C. The supernatant was collected and serotonin release was measured with a serotonin ELISA kit (Labor Diagnostika Nord GmbH & Co, Nordhorn, Germany) according to the manufacturer's instructions.

### 2.10. Measurement of Thromboxane *B*_2_ Formation

Washed platelets (3 × 10^8^/mL) were pre-incubated with or without MAE for 2 min at 37°C in the presence of 1 mM CaCl_2_ and then stimulated with 2.5 *μ*g/mL collagen for 5 min. The reactions were terminated by adding ice-cold 2.5 mM EDTA and 100 *μ*M indomethacin. After centrifugation at 12,000 ×g for 3 min at 4°C, the supernatant was collected and the concentration of TXB_2_ was measured using a TXB_2_ EIA kit according to the manufacturer's protocol.

### 2.11. Immunoblotting

Platelet suspensions (3 × 10^8^/mL) were pre-incubated with MAE or vehicle (0.1% (v/v) DMSO) at 37°C for 2 min. Platelet activation was induced by the addition of 2.5 *μ*g/mL collagen and the reaction was allowed to proceed for 5 min. After terminating the reaction, lysates were then prepared by solubilizing and centrifuging the platelets in sample buffer (0.125 M Tris-HCl, pH 6.8; 2% SDS, 2% *β*-mercaptoethanol, 20% glycerol, 0.02% bromophenol blue, 1 *μ*g/mL phenylmethylsulfonyl fluoride (PMSF), 2 *μ*g/mL aprotinin, 1 *μ*g/mL leupeptin, and 1 *μ*g/mL pepstatin A). Protein concentration was determined using a BCA assay (PRO-MEASURE; iNtRON Biotechnology, Seoul, Republic of Korea). Total cell proteins (30 *μ*g) from the platelet lysate were resolved by 10% SDS-PAGE and transferred to nitrocellulose membranes in transfer buffer (25 mM Tris, pH 8.5; 0.2 M glycine, and 20% methanol). The membranes were blocked in TBS-T containing 5% nonfat dry milk and incubated with primary antibody diluted in a blocking solution. The blots were then incubated with horseradish peroxidase-conjugated secondary antibody. Antibody binding was visualized using enhanced chemiluminescence (iNtRON Biotechnology, Seoul, Republic of Korea).

### 2.12. Assessment of Fibrinogen Binding to Integrin *α*_IIb_ *β*_3_

Fibrinogen Alexa Fluor 488 conjugate binding to washed platelets was quantified by flow cytometry. Briefly, washed platelets (3 × 10^8^/mL) were pre-incubated for 2 min with various concentrations of MAE at room temperature in the presence of 0.1 mM CaCl_2_. The platelets were then stimulated with collagen for 5 min, immediately incubated thereafter with fibrinogen Alexa Fluor 488 (20 *μ*g/mL) for 5 min, and finally fixed with 0.5% paraformaldehyde at 4°C for 30 min. The platelets were pelleted by centrifugation at 2,000 ×g at 4°C and resuspended in 500 *μ*L PBS. Since the activation of integrin *α*_IIb_ *β*_3_ is largely dependent on the generation of Ca^2+^, nonspecific binding of fibrinogen to integrin *α*_IIb_ *β*_3_ was measured by assessing fibrinogen binding in the presence of the calcium chelator EGTA (1 mM). The fluorescence of each platelet sample was analyzed using a FACS Calibur cytometer (BD Biosciences, San Jose, CA, USA), and data were analyzed using CellQuest software (Becton Dickinson Immunocytometry Systems, San Jose, CA, USA).

### 2.13. MAE Effect on Thrombus Formation in Arteriovenous Shunt Thrombosis Model *In Vivo*

The *in vivo* antithrombotic activity of MAE was evaluated in a rat arterio-venous shunt thrombosis model [28]. Rats were given orally administered 400 mg/kg, 200 mg/kg, and 100 mg/kg MAE which were dissolved in 0.25% carboxymethylcellulose (CMC, Sigma, USA) solution at the same time of day for 3 consecutive days by gavage. The shunt thrombosis model was tested 2 h after the last administration. For each test, different batches of six rats were used. After anaesthesia with Urethane (1.25 g/kg i.p) (Sigma, USA), an 8 cm polyethylene tube was inserted between the left jugular vein and the right carotid artery. The saline-filled shunt was assembled by connecting two cannulae with a slightly curved 6 cm long tygon tubing (internal diameter 2 mm) containing a 5 cm long cotton thread (diameter 0.25 mm) which had been scraped with a scalpel blade to render it more thrombogenic. The extracorporeal circulation was maintained for 15 min, during which time a thrombus adheres to the cotton thread. The shunt was then removed and the thread with its associated thrombus was withdrawn and immediately weighed. The thrombus wet weight was determined by subtracting from the value obtained the weight of the dry 5 cm cotton thread determined previously.

### 2.14. Statistics

Data were analyzed with a one-way analysis of variance followed by a *post hoc* Dunnett's test in order to measure statistical significance of the differences observed (SAS Institute Inc., Cary, NC, USA). All data are presented as the mean ± standard error of the mean (SEM). *P* values of 0.05 or less were considered to be statistically significant.

## 3. Results

### 3.1. Chromatographic Separation of *M. alba* Extract

**Figure 1:**
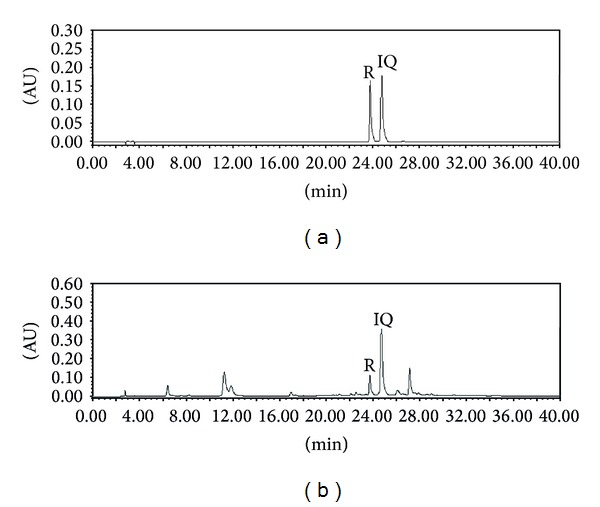
HPLC chromatogram of standard mixture (a) and *Morus alba* leaves extracts at 350 nm. The chromatographic analysis was performed as described in the “Section 2” Identification was based on retention time and UV spectra by comparison with commercial standards. R: rutin; Q: isoquercetin.

As shown Figure 1, high performance liquid chromatographic (HPLC) analysis of MAE revealed rutin and isoquercetin. The MAE contained 2.83 ± 0.15 mg/g for rutin and 8.18 ± 2.4 mg/g for isoquercetin, identified at a retention time of approximately 23.8 min and 24.7 min, respectively.

### 3.2. Inhibitory Effect of MAE on Collagen-Induced Platelet Aggregation

**Figure 2:**
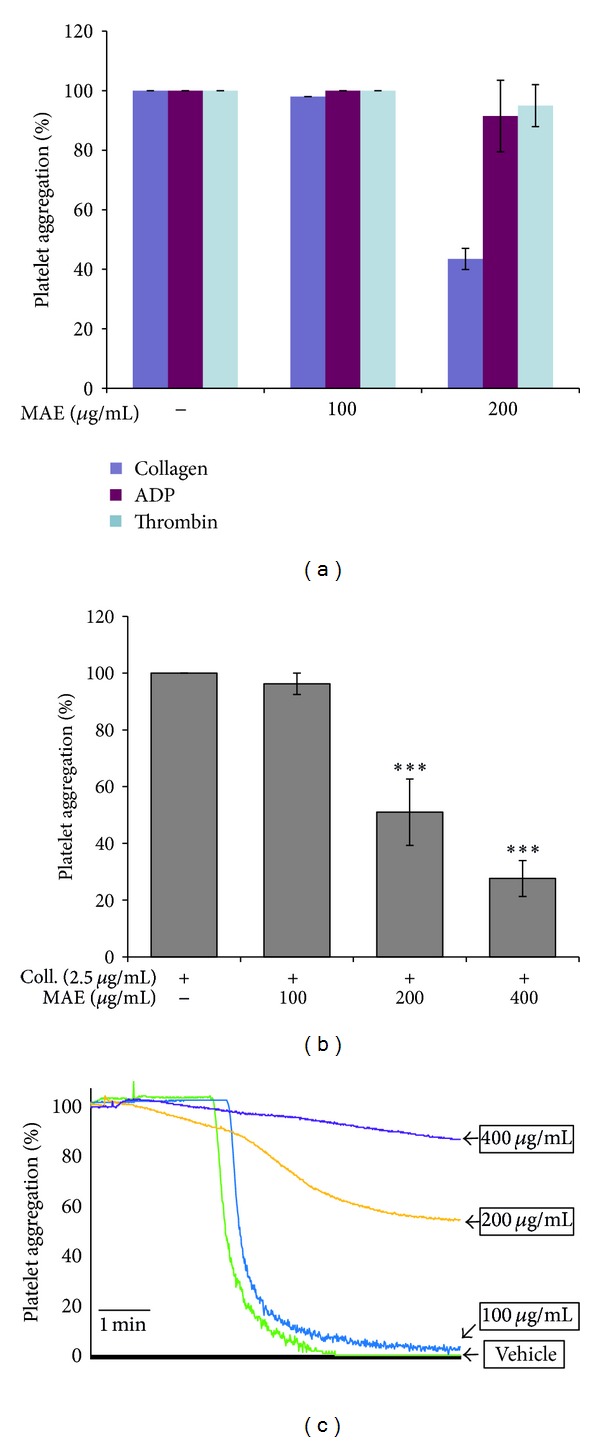
The inhibitory effect of *Morus alba* leaves extracts (MAE) on platelet aggregation induced by collagen. Platelets (3 × 10^8^/mL) were preincubated with or without MAE (100–400 *μ*g/mL) in the presence of 1 mM CaCl_2_ for 2 min at 37°C. The platelet aggregation was then induced by 2.5 *μ*g/mL collagen, 10 *μ*M ADP, and 0.1 U/mL thrombin. The extent of platelet aggregation was measured with an aggregometer. The aggregation reaction was terminated after 5 min, and the percent aggregation rate was calculated. Each graph shows the mean ± SEM of at least four independent experiments. ***P < 0.001 compared to the agonist control.

In the beginning of those studies, we have evaluated whether MAE affected various ligands (ADP-, collagen- and thrombin) induced platelet aggregation. As shown in Figure 2(a), MAE only inhibited collagen-induced platelet aggregation but not in ADP- and thrombin-induced platelet aggregation. In the previous studies [29], we have found that 2.5 *μ*g/mL of collagen, 10 *μ*M of ADP, and 0.1 U/mL of thrombin induced full activation and aggregation of rat platelet. Therefore, we have employed collagen (2.5 *μ*g/mL) as a ligand to induce platelet aggregation in following studies.  Figure 2 shows that MAE inhibited collagen- (2.5 *μ*g/mL) induced platelet aggregation in concentration-dependent manner.

### 3.3. Effect of MAE on Intracellular Calcium Ion Concentration

**Figure 3:**
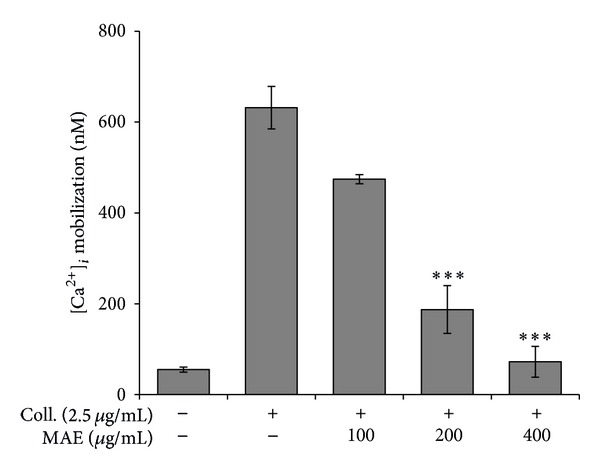
The inhibitory effect of *Morus alba* leaves extracts (MAE) on [Ca^2+^]_*i*_ increased by collagen. Washed platelets (3 × 10^8^/mL) were incubated with a calcium fluorophore (5 *μ*M, Fura-2/AM) and stimulated with collagen (2.5 *μ*g/mL). [Ca^2+^]_*i*_ was then measured as described in Section 2. The results are presented as the mean ± SEM of at least three independent experiments. ***P < 0.001 compared to the agonist control.

It is well known that intracellular calcium ion ([Ca^2+^]_*i*_) takes a pivotal role in the activation of downstream signaling molecules in platelet aggregation induced by a ligand such as collagen. Therefore, we have investigated whether MAE affect the ([Ca^2+^]_*i*_) mobilization induced by 2.5 *μ*g/mL collagen. Collagen induced the massive amount of calcium mobilization by up to 631.7 ± 46.7 nM, which was significantly and concentration-dependently inhibited by MAE (Figure 3).

### 3.4. Effects of MAE on the Granule Release

**Figure 4:**
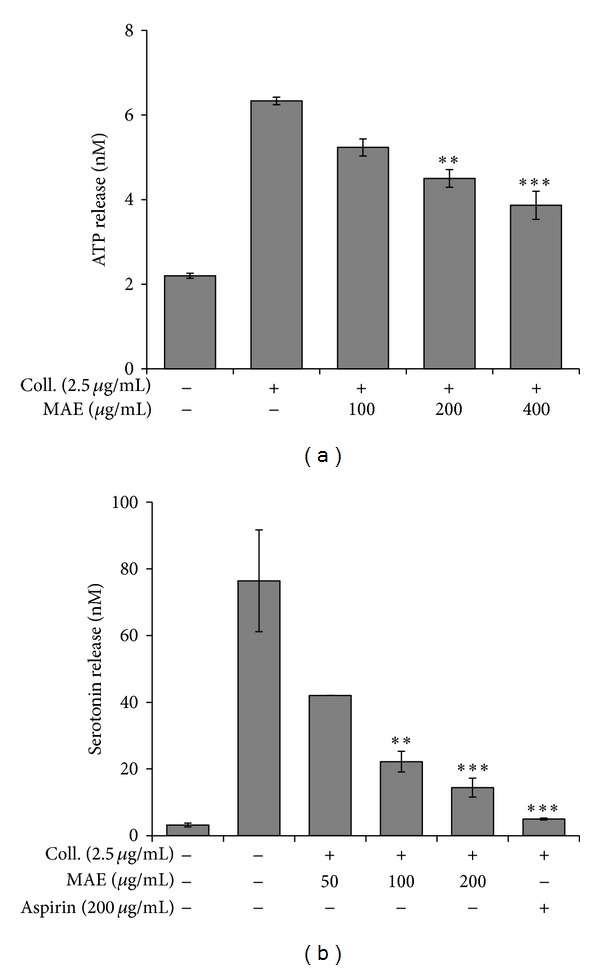
Effects of *Morus alba* leaves extracts (MAE) on granule secretion from the collagen-stimulated platelets. Washed platelets (3 × 10^8^/mL) were pre-incubated with MAE at the indicated concentrations and stirred in an aggregometer for 2 min prior to stimulation with 2.5 *μ*g/mL collagen for 5 min. The reaction was terminated, and an ATP release assay (a) and serotonin release assay (b) were carried out as described in Materials and Methods. Bar graphs show the mean ± SEM of at least four independent experiments. **P < 0.01 and ***P < 0.001 compared to the agonist control.

In the following studies, we have examined whether the extract modulated the secretion of granule contents such as ATP and serotonin which can activate platelets themselves acting as autacoids. Collagen (2.5 *μ*g/mL) considerably induced ATP release from dense granule by 3-fold in comparison with resting platelets. As depicted in Figure 4(a), MAE dose-dependently repressed collagen- (2.5 *μ*g/mL) induced ATP release. In order to confirm the MAE's effect on dense granule, we have chosen another marker molecule serotonin in dense contents. As expected, MAE potently and concentration-dependently inhibited serotonin release which was induced by 2.5 *μ*g/mL collagen (Figure 4(b)).

### 3.5. Effects of MAE on TXA2 Formation

**Figure 5:**
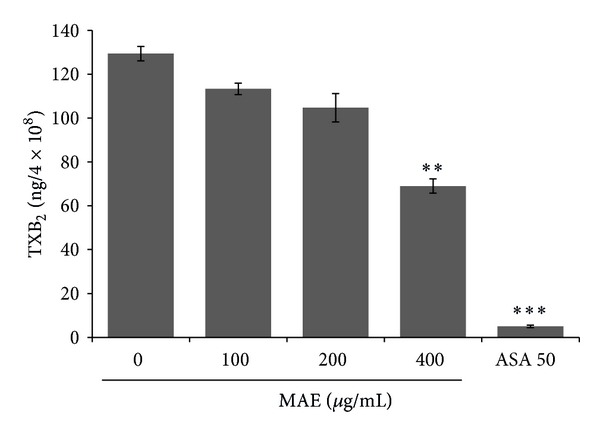
Effects of *Morus alba* leaves extracts (MAE) on TXA2 formation from the collagen-stimulated platelets. Washed platelets (3 × 10^8^/mL) were pre-incubated with MAE at the indicated concentrations and stirred in an aggregometer for 2 min prior to stimulation with 2.5 *μ*g/mL collagen. The reaction was terminated at 5 min, and TXA2 formation was performed as described in Materials and Methods. Bar graphs show the mean ± SEM of triplicate independent experiments. **P < 0.01 and ***P < 0.001 compared to the agonist control.

We next investigated whether MAE affected TXA2 formation in collagen-activated platelets. TXA2 is another marker molecule in the initial activation of ligand binding to cognate receptor. In addition, it acts as an agonist against platelet own receptor, which, therefore, is named “autocoid.” TXB2 is stable metabolite of TXA2 and thus TXB2 formation was measured.  Figure 5 displayed that MAE significantly inhibited TXA2 formation in collagen-activated platelets.

### 3.6. Effects of MAE on the Phosphorylation of MAPKs and Akt

We next investigated whether MAE affected the phosphorylation of mitogen-activated protein kinase (MAPK), including extracellular-signal regulated kinase 1/2 (ERK1/2), p38 MAPK, and c-Jun N-terminal kinase (JNK). In previous studies [26, 30], we have shown that all these MAPKs were expressed and phosphorylated by ligands such as ADP and collagen. Our immunoblot analysis revealed that MAE potently inhibited collagen-induced ERK phosphorylation but marginally repressed collagen-induced JNK phosphorylation (Figure 7). However, collagen-induced p38 MAPK phosphorylation was not affected by the extract treatment. In addition, ligand binding to cognate receptor has been shown to activate phosphatidylinositol 3-kinase (PI3K) and Akt, which target the glycogen synthase kinase (GSK) 3*β* as an Akt effecter. Therefore, we have investigated whether MAE modulated collagen-induced Akt activation. As depicted in Figure 7(b), MAE potently and concentration-dependently suppressed Akt phosphorylation which was activated by collagen.

### 3.7. Effects of MAE on the Integrin *α*_IIb_ *β*_3_ Activation

In order to complete platelet aggregation stably, outside-in signaling pathway should be activated, as determined with fibrinogen binding to active integrin *α*_IIb_ *β*_3_.

**Figure 6:**
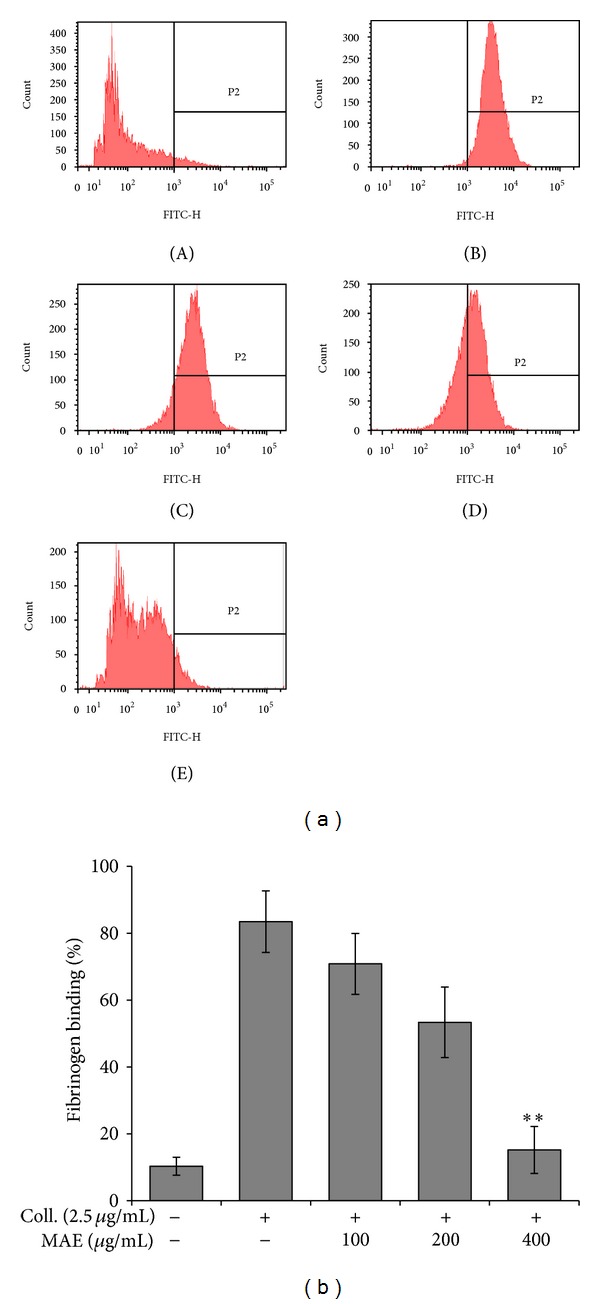
Effects of *Morus alba* leaves extracts (MAE) on fibrinogen binding to integrin *α*_IIb_ *β*_3_ in ADP-activated platelets. The inhibitory effects of MAE on fibrinogen binding to integrin *α*_IIb_ *β*_3_ in collagen-activated platelets were measured by flow cytometry (A). Washed platelets (3 × 10^8^/mL) were pretreated with vehicle (DMSO) or MAE at concentrations ranging from 100 *μ*g/mL to 400 *μ*g/mL. Collagen was then incubated with human fibrinogen labeled with Alexa Fluor 488 (20 *μ*g/mL) for 5 min. The cells were subsequently fixed with 0.5% paraformaldehyde at 4°C for 30 min. Graphs showing fluorescent intensity present the data from one experiment but are representative of four independent trials. Data are expressed as the mean fluorescence intensity (MFI) of fibrinogen-positive platelets. Each graph presents the results expressed as percent of gated (a). **P < 0.01 compared to the agonist control.

The influence of MAE on integrin *α*_IIb_ *β*_3_ activation was studied in collagen-stimulated platelets. As shown in Figure 6, collagen (2.5 *μ*g/mL) increased the fibrinogen binding to active integrin *α*_IIb_ *β*_3_, whereas resting platelets did not activate the integrin.

The plant extracts reduced collagen- (2.5 *μ*g/mL) induced fibrinogen binding to integrin *α*_IIb_ *β*_3_ in concentration-dependent manner (Figure 6).

### 3.8. Effect of MAE on Arteriovenous Shunt Thrombosis

The AV-shunt thrombosis model has been commonly used to assess antithrombotic effects. Compared to the control, a 3-day oral treatment with MAE decreased thrombus weight in the rat arteriovenous shunt thrombosis model. As shown in Figure 8, after oral administration of 100, 200, and 400 mg/kg/day, MAE significantly and dose-dependently decreased the thrombus weight by 52.3% (*P* < 0.001), 28.3% (*P* < 0.01), and 19.1% (*P* < 0.05) compared to vehicle control, respectively. Rivaroxaban which was used as a positive control is an oral anticoagulant for the prevention and treatment of thrombosis mediated conditions [31]. Rivaroxaban 5 mg/kg/day was different from control (*P* < 0.001) and MAE 100 mg/kg/day (*P* < 0.05), but not from MAE 200 mg/kg/day and 400 mg/kg/day.

## 4. Discussion

These results show that *in vitro* the ethanol extract of *M. alba* L. leaf (MAE) inhibits collagen-induced platelet aggregation in a concentration-dependent manner. In order to elucidate the mechanism of inhibitory activities of MAE extract, we have analyzed downstream signaling as follows intracellular calcium concentration, dense granule secretions, protein phosphorylations (e.g., MAPKs and Akt), and integrin signaling. In addition, we next investigated whether this efficacy of MAE *in vitro* is implemented in *in vivo* application. That is, the extract given orally to rats for 3 days dose-dependently decreased thrombus weight in a common model of arterial thrombosis. To the best of our knowledge this is the first report to demonstrate the effects on platelet aggregation and thrombosis.

Upon the ligand binding to platelet membrane receptors, [Ca^2+^]_*i*_ plays an important role in the initial activation of platelet and subsequent platelet aggregation. Upregulation of [Ca^2+^]_*i*_ is due to both calcium influx from extracellular fluid and calcium mobilization from intracellular pools, such as dense tubular system and mitochondria. Although unknown is the source of [Ca^2+^]_*i*_, collagen dramatically escalates intracellular calcium concentration which is significantly and concentration-dependently reduced by MAE. In addition, we did not determined whether MAE modulate either calcium influx in platelet membrane or inositol-1,4,5-trisphosphate (IP3) receptor on platelet organelles. However, MAE potently blocked collagen-induced [Ca^2+^]_*i*_ upregulation which is at least main cause of antiplatelet activity.

Mammalian platelets devoid of nucleus and basically transcriptional and translational activities are limited. Although platelet transcription factors such as nuclear factor kappa B (NF-*κ*B) are some reported [32, 33], it is largely understood that new functional protein synthesis is limited. Therefore, granule substances take a pivotal role in the platelet activation and aggregation due to its biological and functional significance. For example, dense granules enclosed ATP, ADP, serotonin and Ca^2+^. They act as an autacoid (e.g., self-activator) or an important modulator in the downstream signaling. Therefore, the MAE's antiplatelet activity might be due to the potent inhibition of granule secretion (i.e., Ca^2+^, ATP, and serotonin) (Figure 9).

The activation of integrin *α*_IIb_ *β*_3_ is plays an important role in the final step of platelet activation and aggregation, which is named as inside-out signaling. That is, the phosphorylation of integrin *α*_IIb_ *β*_3_ is able to bind to another phosphorylated integrin *α*_IIb_ *β*_3_, which is mediated via fibrinogen-like bridge network. Finally, strict and stable blood thrombus is being formed. Therefore, screening of pharmacological inhibitors is widely being investigated for antiplatelet and antithrombotic agents. At the moment, we insisted that MAE could be developed as antiplatelet or antithrombotic agents or functional food.

On the other hand, the phosphorylation of signaling molecules, including MAPKs (i.e., ERK1/2, p38 MAPK, and JNK) and PI3K/Akt is also crucial phase in outside-in and inside-out signaling of platelet aggregation. In our results, the collagen-induced phosphorylation of ERK and JNK was inhibited by MAE but not p38 MAPK. Although the role of ERK and JNK in platelet physiology is not clear, there are several evidences that they are involved in the activation of integrin *α*_IIb_ *β*_3_ and PLA_2_/TXA_2_ pathways [34, 35]. In addition, MAE blocked collagen-induced TXA2 production (Figure 5). This indicated that MAE blocked MAPK-integrin *α*_IIb_ *β*_3_ and PLA_2_/TXA_2_ routs and thus displayed potent antiplatelet activity.

**Figure 7:**
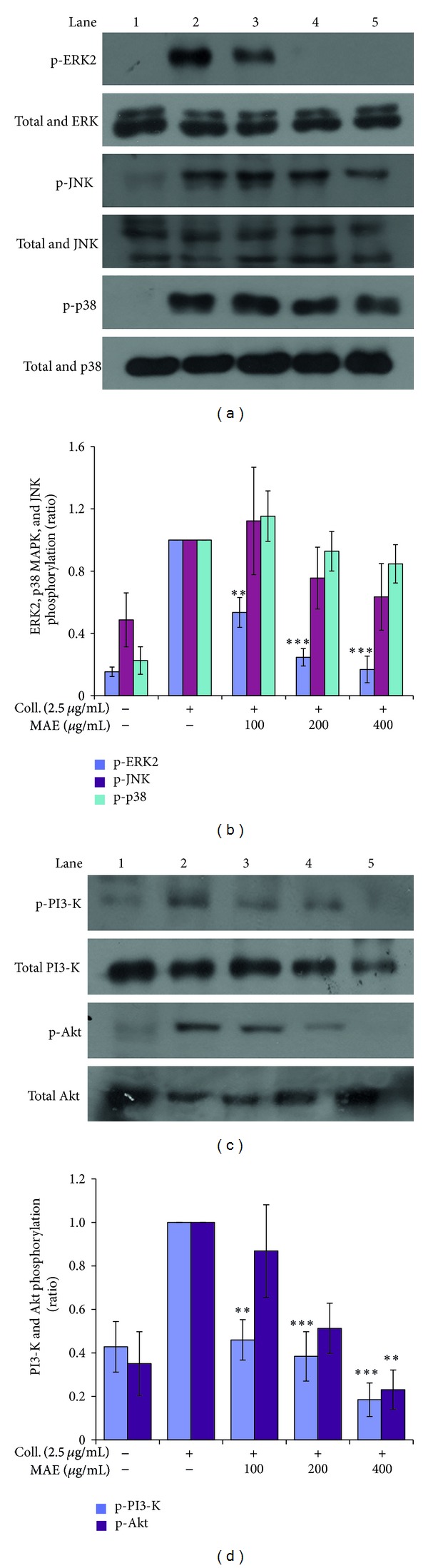
Effects of *Morus alba* leaves extracts (MAE) on collagen-induced phosphorylation of MAPKs, PI3K, and Akt. Washed platelets (3 × 10^8^/mL) were pre-incubated for 2 min with vehicle or MAE at the indicated concentration. The platelets were then stimulated with 10 *μ*M ADP for 5 min at 37°C. After terminating the reactions, total cell proteins were extracted. The proteins were separated by SDS-PAGE and transferred onto nitrocellulose membranes. The membranes were then probed with antibodies against phospho-p44/42, p44/42, phospho-p38, p38, phospho-SAPK/JNK, and *β*-actin ((a), (b)) and phospho-PI3K, phospho-Akt, PKA*α*/*β*/*γ* cat, and *β*-actin ((c), (d)). Antibody binding was visualized by chemiluminescence. All immunoblots are representative of three or four independent experiments. **P < 0.01 and ***P < 0.001 versus vehicle control.

**Figure 8:**
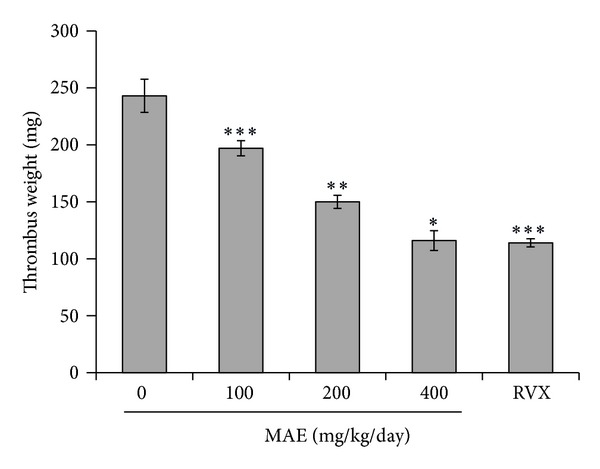
Effect of *Morus alba* leaves extracts (MAE) on thrombus formation in rats. The procedures for the AV-shunt model are described in the Section 2. Thrombus weight in an arteriovenous shunt was measured at 2 h after administration of 3-day treatment with 0.25% carboxymethylcellulose solution (control), ethanol extract of *Morus alba* L. leaves (MAE) 100, 200 or 400 mg/kg/day, and positive control (rivaroxaban, RVX) 5 mg/kg/day. Data are shown as mean ± S.D. *P < 0.05, **P < 0.01, ***P < 0.001 versus vehicle control.

**Figure 9:**
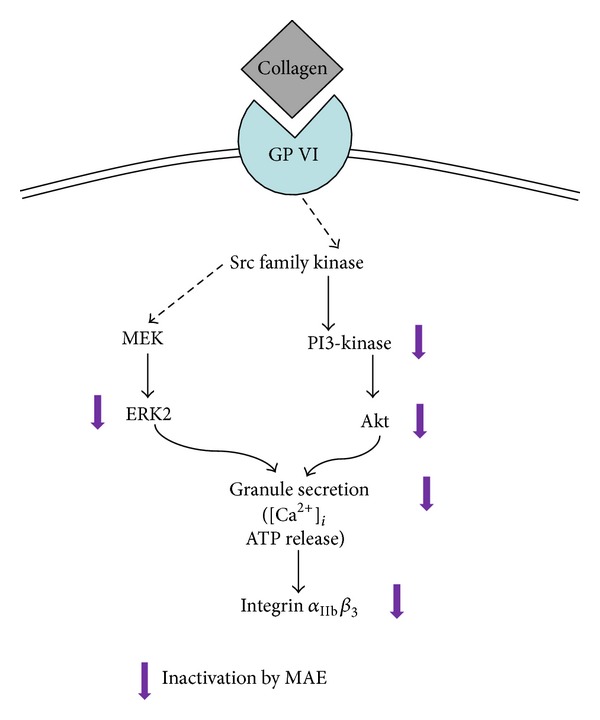
The summary of inhibitory effect of MAE in collagen-induced platelet aggregation. GP VI: glycoprotein VI; MEK1 mitogen-activated protein kinase kinase; ERK2, extracellular-regulated kinase 2.

On the other hand, PI3-K/Akt pathway plays important role in the platelet activation and aggregation. The receptor activation leads to the phosphorylation of PI3-K and subsequently activation and phosphorylation of Akt. Moreover, it is reported that signaling routes of PI3-K are involved in the transient activation of integrin *α*_IIb_ *β*_3_. As shown in Figure 7(b), MAE significantly inhibited both PI3-K and Akt phosphorylation. However, at the moment, direct inhibition of MAE on either PI3-K or PI3-K and Akt is not clear, which remains to be elucidated in the future.

Blood flow disturbances at sites of atherosclerotic plaque rupture promote platelet activation and arterial thrombus formation [36, 37]. In the present study, MAE significantly inhibited collagen-induced platelet aggregation *in vitro* and rat carotid artery thrombus formation *in vivo*. Therefore, MAE has potential to prevent thrombotic or cardiovascular disease.

MAE also inhibited serotonin and ATP secretion in a concentration-dependent manner. Serotonin and ATP can cause vasoconstriction of interarterial coronary collateral vessels, and they suggest that platelet activation in coronary arteries that are linked to collateral vessels has the potential to cause collateral vasoconstriction, thereby compromising blood flow to the dependent myocardium [38–40]. Therefore MAE might be used for prevention or treatment of myocardial infarction.

The butanol-soluble fraction from MAE showed the strongest inhibitory effect against collagen-induced platelet aggregation when compared with other fractions, hexane, ethyl acetate and water fractions of MAE. The finding indicates that active principles are medium polar, suggesting that alcoholic extract rather than water extract such as tea or beverage might be beneficial for cardiovascular.

In this study, from MAE we identified two quercetin glycosides, rutin and isoquercetin, which have been reported to be major components of *M. alba* leaf along with quercetin as major components in *M. alba* leaf [18, 41].

We also further evaluated the inhibitory effects of rutin and isoquercetin at high concentration of 400 *μ*g/mL on collagen-induced platelet aggregation, but they slightly inhibited (Data not shown). Since their aglycone, quercetin, is known to inhibit strongly on platelet aggregation rabbit platelets induced by collagen, ADP, or arachidonic acid [26, 42], we also investigated its existence using its commercial standard but not identified on HPLC chromatogram; a standard quercetin was retained approximately at 34.6 min (data not shown), but any traceable peak was not appeared near the retention time as shown Figure 1. Further study would be required in order to identify the active compound(s).

## 5. Conclusions

In *in vitro* assays using freshly isolated rat platelets, the MAE showed significant inhibition of collagen-induced platelet aggregation, and these effects were also associated with reduced [Ca^2+^]_*i*_, ATP and serotonin secretion, and integrin *α*_IIb_ *β*_3_. In accordance with these enhanced *in vitro* antiplatelet activities, the extract showed enhanced antithrombotic effects in *in vivo* arterial thrombosis model. Thus, these results suggest that MAE can be a good candidate to an antiplatelet and antithrombotic agent.
